# Saikosaponin-d alleviates hepatic fibrosis through regulating GPER1/autophagy signaling

**DOI:** 10.1007/s11033-021-06807-x

**Published:** 2021-10-29

**Authors:** Yirong Chen, Renye Que, Na Zhang, Liubing Lin, Mengen Zhou, Yong Li

**Affiliations:** 1 Department of Gastroenterology, Shanghai Municipal Hospital of Traditional Chinese Medicine, Shanghai University of Traditional Chinese Medicine, No. 274 Zhijiang Road, Shanghai, 200071 China; 2 Department of Gastroenterology, Shanghai TCM Integrated Hospital, Shanghai University of Traditional Chinese Medicine, Shanghai, 200082 China

**Keywords:** Saikosaponin-d, Autophagy, GPER1, G15, Liver fibrosis

## Abstract

**Background:**

Hepatic fibrosis is the final pathway of chronic liver disease characterized by excessive accumulation of extracellular matrix (ECM), which eventually develop into cirrhosis and liver cancer. Emerging studies demonstrated that Saikosaponin-d (SSd) exhibits a protective role in liver fibrosis. However, the mechanism underlying anti-liver fibrosis of SSd in vivo and in vitro remains unclear.

**Methods and results:**

Transforming growth factor (TGF)-β and carbon tetrachloride (CCl_4_) were used for creating liver fibrosis model in vitro and in vivo, respectively. The role of SSd in regulating liver fibrosis was assessed through Sirius red and Masson staining, and IHC assay. We found that SSd attenuated remarkably CCl_4_-induced liver fibrosis as evidenced by decreased collagen level, and decreased expression of fibrotic markers Col 1 and α-SMA. Meanwhile, SSd repressed autophagy activation as suggested by decreased BECN1 expression and increased p62 expression. Compared with HSCs from CCl_4_-treated group, the primary HSCs from SSd-treated mice exhibited a marked inactivation of autophagy. Mechanistically, SSd treatment enhanced the expression of GPER1 in primary HSCs and in TGF-β-treated LX-2 cells. GPER1 agonist G1 repressed autophagy activation, whereas GPER1 antagonist G15 activated autophagy and G15 also damaged the function of SSd on suppressing autophagy, leading to subsequent increased levels of fibrotic marker level in LX-2 cells.

**Conclusions:**

Our findings highlight that SSd alleviates hepatic fibrosis by regulating GPER1/autophagy pathway.

## Introduction

Liver fibrosis is an intermediate stage in the progression of chronic liver diseases to cirrhosis, characterized by a large number of fibrous hyperplasia, excessive production and deposition of extracellular matrix (ECM) in the liver [1, 2]. Revealing the underlying mechanism of liver fibrosis is of great significance for the prevention and treatment of liver cirrhosis [3]. Although there is no safe and effective drug for liver fibrosis, the efficacy of traditional Chinese medicine and natural products has been increasingly emphasized in treating liver fibrosis.

Radix bupleuri is a common herbal medicine in China for treating chronic inflammation, pain, and fever [4]. Saikosaponin d (SSd) is the main active monomer of Radix bupleuri, and possesses several pharmacological activities including anti-inflammation [5], anti-tumor [6], antimicrobial [7], lowering blood pressure [8], and immunoregulation [9]. SSd accelerates apoptosis and represses proliferation of HSCs through regulating phosphorylation of extracellular signal-regulated kinase 1/2 (ERK1/2) and p38 [10]. SSd also possesses an anti-proliferative role in T lymphocyte through repressing NF-AT, NF-κB, and AP-1 signaling [11]. Given the critical role of inflammation in liver fibrosis, it is possible that SSd possesses an anti-fibrotic effect. Additionally, emerging evidence has shown that SSd contributes to alleviate hepatic cell injury induced by CCl_4_, alcohol and high fat [12]. In previous studies, we demonstrated that SSd attenuates CCl_4_-induced acute liver injury in mice through repressing oxidative stress and subsequent NLR family pyrin domain containing 3 (NLRP3) inflammasome pathway [13, 14]. However, the roles of SSd in liver fibrosis and the potential mechanism in this process remain unclear.

Autophagy is a highly conserved process that contributes to maintain cell homeostasis by degrading protein aggregates, impaired organelles, and cellular debris [15, 16]. The aberrant activation of autophagy is related to various pathological processes such as hepatic injury, inflammation [17], tumors [18], and fibrosis [19]. Autophagy inhibition by autophagy-related protein (ATG)-7 knockout leads to the aggregation of abnormal mitochondria and p62/SQSTM1-containing protein in hepatic cells, and thus results in a liver injury [20]. These data indicate that autophagy plays an important role in protecting liver cells from injury. However, the role of autophagy in liver fibrosis remains controversial. Lee et al., demonstrated that autophagy inhibition by chloroquine attenuates carbon tetrachloride (CCl_4_)-induced hepatic fibrosis by repressing the activation of HSCs [21]. Autophagy improves hepatic fibrosis by protecting LSECs (liver sinusoidal endothelial cells) from oxidative stress [22]. Inversely, several studies showed that autophagy facilitates liver fibrossi by activating HSCs in thioacetamide (TAA) or CCl_4_-treated mice [23, 24].

In the study, we explored the anti-liver fibrosis effects of SSd on transforming growth factor (TGF)-β-treated HSCs in vitro or CCl_4_-induced liver fibrosis in vivo. Also, we assessed the role of SSd in regulating autophagy in HSCs. The current data demonstrate that SSd treatment contributes to alleviate liver fibrosis through regulating GPER1/autophagy pathway.

## Materials and methods

### CCl_4_-induced hepatic fibrosis

Animal experiments were conducted with approval from the Experimental Animal Committee of Shanghai Municipal Hospital of Traditional Chinese Medicine, Shanghai University of Traditional Chinese Medicine, and all experiments were completed in accordance with the ARRIVE guidelines as described previously [25] to minimize the mice number and suffering. The C57 mice (male, 25–30 g, 6–8 w) were obtained from Beijing Vital River Laboratory Animal Technology Co., Ltd. (Beijing, China). CCl_4_ (56-23-5) was obtained from Sinopharm Chemical Reagent Co., Ltd. (Shanghai, China) and olive oil was obtained from Solarbio (Beijing, China). All mice were randomly divided into three groups (n = 5 of each group): (1) control group, the control mice were injected with commensurable olive twice a week for 6 weeks, and CCl_4_-induced group and SSd treatment group mice were intraperitoneal injected CCl_4_ (0.8 ml/kg) twice a week for 6 weeks. At the same time, SSd treatment group mice were administered SSd (19810421, Wako Pure Chemical Industries, Ltd, Japan) daily, by intraperitoneal injection at a dosage of 2.0 mg/kg for 6 weeks.

### Primary HSCs isolation and cell culture

HSCs were isolated from male C57 mice according to previously reports [26]. Briefly, the mice were euthanised by cervical dislocation after anesthetizing with a mixture of Ketamine (75 mg/kg) and Medetomidine (1 mg/kg), and then the liver was perfused by portal vein using Pronase-E (Sigma-Aldrich) and collagenase (A005275, Sangon Biotech, Shanghai, China). The HSCs population was separated through density centrifugation with Nycodenz solution (Axis Shield POC, Oslo, Norway). Isolated HSCs were cultured in DMEM/F12 (Sigma) supplemented with 20% FBS, 1% penicillin–streptomycin in the humidified 5% CO_2_ incubator at 37 °C.

Human Hepatic Stellate Cells LX-2, obtained from Merck Millipore (SCC064, Temecula, California, USA), were cultured in DMEM supplemented with 2% FBS and 1% penicillin–streptomycin at 37 °C in a humidified atmosphere containing 5% CO_2_. LX-2 cells were treated with 5 ng/ml TGF-β (Beyotime, Shanghai, China) for 24 h.

### Sirius red and Masson staining

The liver tissues were fixed with 10% formaldehyde, embedded in paraffin and cut into 5-μm sections. Then the sections were stained in 1% Sirius red solution or Masson solution for 1 h or Masson solution for 5 min, observed using a microscope (Olympus, Tokyo, Japan).

### Immunohistochemistry (IHC)

The liver tissues were fixed with buffered formalin (10%), embedded in paraffin, and cut into 4 µm-thick sections. The slices were incubated with citrate buffer (pH 6.0) for 5 min at 108 °C, pretreated with 3% hydrogen peroxide (H_2_O_2_) for 15 min at room temperature and washed with PBS. Then, the slices were incubated with normal goat serum for 20 min, followed by incubation with primary antibody against α-SMA (1:500, ab108424, Abcam), Col1α1 (1:50, PA5-36227, Invitrogen) and BECN1 (1:100, ab210498, Abcam) overnight at 4 °C. Besides, the slices were incubated with secondary antibody (1:500, horseradish peroxidase-conjugated anti-rabbit IgG) and the reaction products were visualized with DAB solution.

### Immunofluorescence (IF)

LX-2 cells were fixed with 4% paraformaldehyde for 0.5 h at room temperature and followed by washing three times in PBS. Next, cells were blocked with PBS supplemented 5% FBS and 0.2% Triton X-100 for 1 h at room temperature, and then incubated with anti-LC3 antibody (1 µg/ml; ab48394; Abcam) overnight at 4 °C. After washing, the slides were incubated with goat anti-rabbit antibody (1:2000; A27039; Invitrogen) for 2 h at room temperature without light. The slides were washed three times in PBS and stained with DAPI (Solarbio) to visualize the nucleus. Finally, the slides were washed, mounted with glycerin, and analyzed using a confocal microscopy (Thorlars, Newtown, New Jersey, USA).

### Quantitative real-time pCR (qPCR)

Total RNA was separated with Trizol reagent (Sigma-Aldrich, St. Louis, MO, USA) as instructed by the manufacturer. Reverse transcriptional PCR was performed using the iScripe™ cDNA Synthesis kit (Bio-Rad). qPCR was performed on ABI 7500-Fast Real-Time PCR System (Applied Biosystem, Foster City, CA, USA) with LightCycler 480 SYBR Mix (Roche). The fold changes of RNA transcripts were calculated by the 2^−ΔΔCt^ method and the β-actin was used as a reference gene. COL1A1 F ACGGCTCAGAGTCACCCA; COL1A1 R CCTCCGGTTGATTTCTCATCATA; α‐SMA F TCCCTTGAGAAGAGTTACGAGTT; α‐SMA R ATGATGCTGTTGTAGGTGGTT; ERα, F AGTGAAGCCTCAATGATGGG, ERα, R CAAAGATCTCCACCATGCCT; ERβ, F CTACTGAACGCGGTGACAGA, ERβ, R CGTGTCAGCATTCAGCATCT; GPER1, F CCATCATCGGCCTGTGCTAT, GPER1, R GAAGACAAGGACCACTGCGA; β-actin F CTTAGTTGCGTTACACCCTTTCTTGA; β-actin R CTGTCACCTTCACCGTTCCAGTTT.

### Western blot analysis

The LX-2 cells or liver tissues from CCl_4_-induced or SSd treatment mice were lysed using RIPA Buffer (Solarbio, Beijing, China), and the total protein concentration was detected by BCA protein assay kit (Cell Signaling Technology, Danvers, MA, USA). The proteins were separated by 10% SDS-PAGE, and then transferred to PVDF membranes (Merck Millipore, Billerica, MA, USA). After blocking with 5% nonfat milk in TBST, the membranes were incubated with primary antibodies against p62 (1:2000, ab109012, Abcam), LC3 (2 µg/ml, ab128025, Abcam), GPER1 (1:1000, ab260033, Abcam), α-SMA (1:1000, ab108424, Abcam) and β-actin (1:5000, ab6276, Abcam) overnight at 4 °C. The blots were incubated with HRP-conjugated secondary anti-rabbit (1:5000) for 1 h at room temperature. Lastly, Immunoreactivities were visualized by chemiluminescence using the ECL kit (Pierce, Rockford, IL, USA). The intensity of protein bands on the Western blot image was quantified using ImageJ software.

### Cell vitality

Cell Counting Kit-8 (CCK-8) assay was applied to measure LX-2 cell viability using CCK-8 kit (Solarbio, Beijing, China). Cells were plated at 4 × 10^3^ cells per well in 96-well plates and treated with 5 ng/ml TGF-β and SSd (5 μM), G15 (100 nM) or Rapa (100 nM) for 24 h. After that, the cells were incubated with 10 µl CCK-8 for another 2 h at 37 °C. The absorbance was measured at 450 nm with a microplate reader (Bio-Rad, Hercules, CA, USA).

### Statistical analysis

Data are expressed as mean ± SEM. All statistics were carried out with SPSS 13.0 (IBM, Armonk, NY, USA). The difference between two groups was compared using two-tailed student’s *t*-test, or one-way analysis of variance (ANOVA) followed by the Scheffé test. P‐values less than 0.05 were considered statistically significant.

## Results

### SSd alleviated hepatic fibrosis in CCl_4_-treated mice

**Fig. 1:**
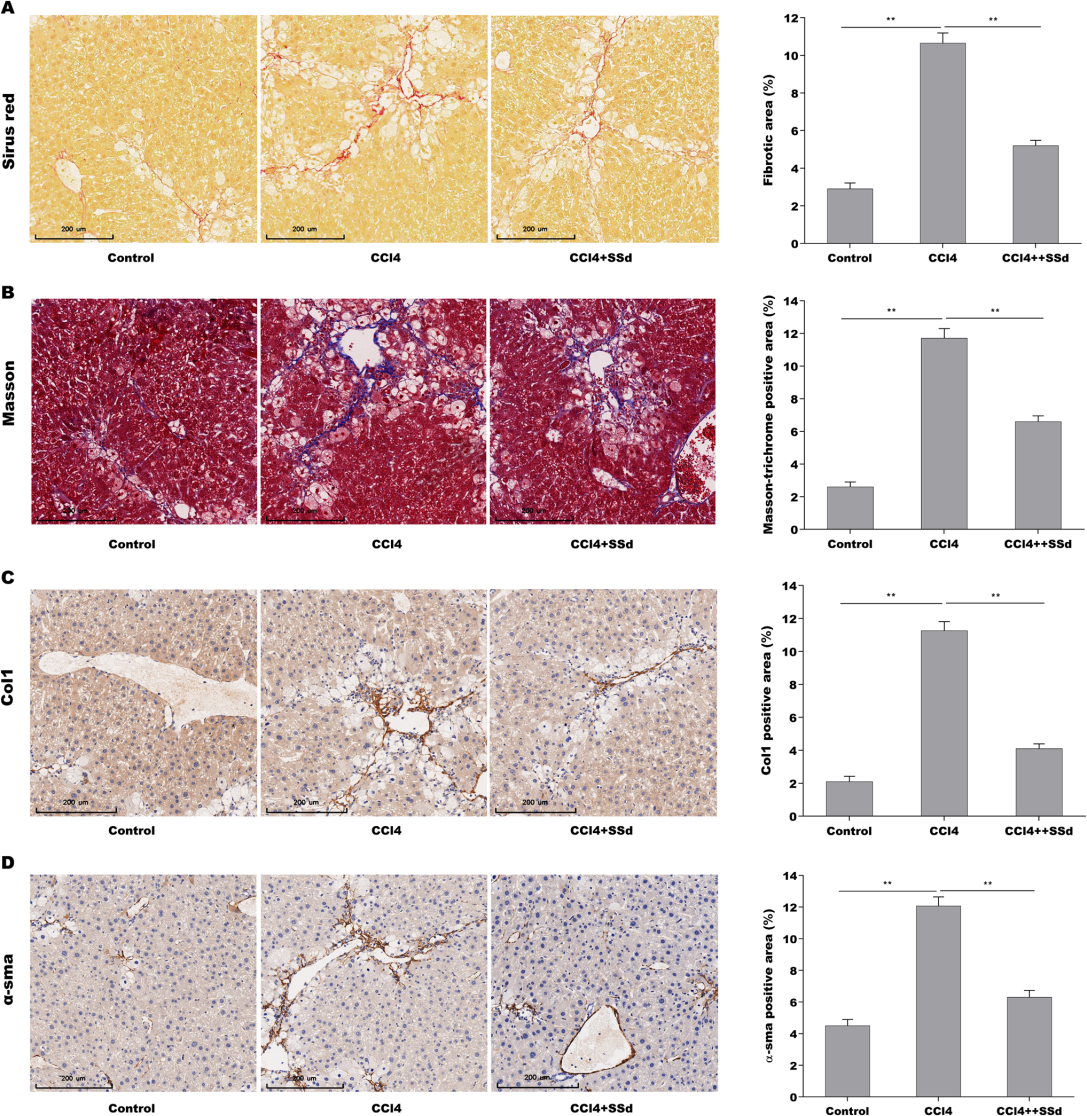
SSd alleviated hepatic fibrosis in CCl_4_-treated mice. Sirius red (**A**) and Masson staining (**B**) for liver tumors in control mice (n = 5), CCl_4_-treated mice (n = 5) and CCl_4_-SSd-treated mice (n = 5). IHC for Col1α1 (**C**) and α-SMA (**D**) levels in liver tumors of control mice (n = 5), CCl_4_-treated mice (n = 5) and CCl_4_-SSd-treated mice (n = 5). Scale bars = 200 μm for Sirius red, Masson and IHC. Below, five images of each liver and five livers from different mice were quantified for each group. **p < 0.01. (Color figure online)

The role of SSd in regulating hepatic fibrosis was first evaluated in CCl_4_-induced hepatic fibrosis mice. The results from Sirius red and Masson staining showed that SSd repressed significantly CCl_4_-induced increase of collagen level in liver tissues (Fig. 1A, B). Immunohistochemical (IHC) analysis further revealed that the α-SMA and Col 1 protein expression was increased markedly in fibrotic liver tissues compared with control liver tissues (Fig. 1C, D). The effect of SSd on alleviating hepatic fibrosis was next evaluated in vivo. As expected, SSd treatment ameliorated remarkably CCl_4_-induced hepatic fibrosis in mice, as suggested by decreased expression of α-SMA and Col 1 (Fig. 1C, D). These results verify the protective role of SSd in repressing hepatic fibrosis.

### SSd repressed autophagy activation in CCl_4_-treated mice

**Fig. 2:**
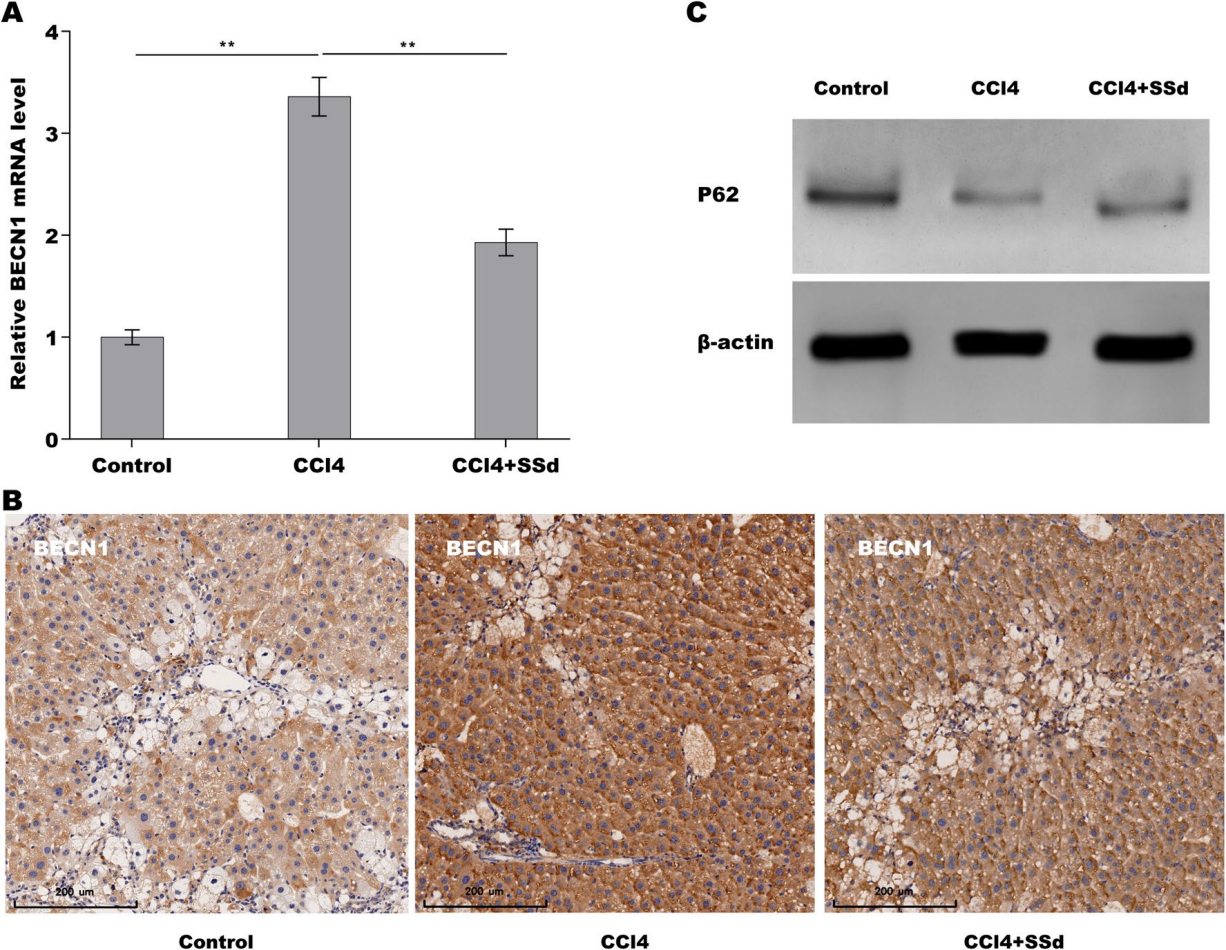
SSd repressed autophagy activation in CCl_4_-treated mice. qPCR analysis (**A**) and IHC (**B**) of BECN1 expression level in CCl_4_ mice treated with or without SSd (0.8 ml/kg). Scale bars = 200 μm for IHC. **B** Western blot analysis for p62 protein expression level in CCl_4_ mice treated with or without SSd (0.8 ml/kg). **p < 0.01

Inactivation of HSCs autophagy contributes to repress HSCs activation and subsequent liver fibrosis [27]. Then we investigated whether autophagy activation was increased in fibrotic liver tissues and SSd treatment repressed the activation of autophagy. As shown in Fig. 2A, the mRNA level of autophagy marker BECN1 was increased in fibrotic liver tissues compared with control tissues, whereas SSd treatment repressed CCl_4_-induced upregulation of BECN1 expression. IHC assay also showed that SSd suppressed CCl_4_-induced protein expression of BECN1 in liver tissues (Fig. 2B). p62/SQSTM1 (p62) is known as autophagy substance, and the downregulation of p62 expression is an indicator of autophagy activation [28]. Figure 2C showed that p62 level was decreased markedly in fibrotic liver tissues compared with control tissues, whereas SSd treatment partially enhanced p62 expression in liver tissues, indicating that SSd repressed autophagy activation in fibrotic livers.

### SSd repressed CCl_4_-induced autophagy activation of HSCs in vivo

**Fig. 3:**
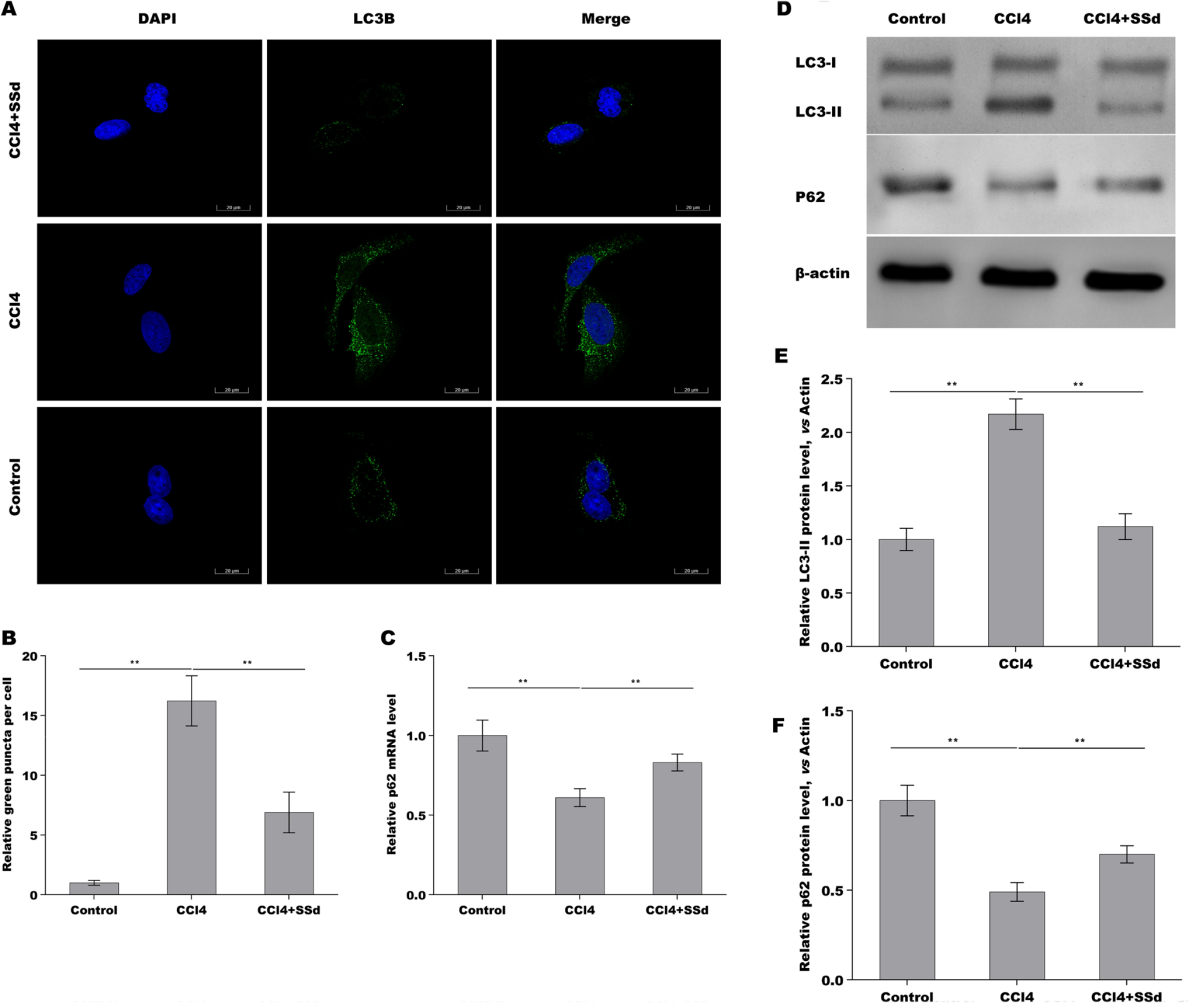
SSd repressed CCl_4_-induced autophagy activation of HSCs in vivo. **A**, **B** LC3B expression was detected in primary HSCs from hepatic tissues in control mice, CCl_4_ mice and SSd-treated mice by IF assay. **C** qPCR analysis of p62 expression level in primary HSCs from hepatic tissues in control mice, CCl_4_ mice and SSd-treated mice. **D**–**F** Western blot analysis for LC3 (**D**, **E**) and p62 (**D**, **F**) protein expression level in primary HSCs from hepatic tissues in control mice, CCl_4_ mice and SSd-treated mice. **p < 0.01

We next explored whether SSd repressed HSCs autophagy activation because HSCs is a main cell type responsible for liver fibrosis [29]. For this purpose, primary HSCs were acquired from hepatic tissues in control mice, CCl_4_ mice, and SSd-treated mice, respectively, and autophagy activation was assessed. As shown in Fig. 3A and B, the HSCs from CCl_4_ mice exhibited a marked upregulation of LC3 puncta (green) compared with the HSCs from control mice, while SSd treatment inhibited significantly CCl_4_-induced LC3 puncta in HSCs. The mRNA level of p62 was reduced in the HSCs from CCl_4_ mice compared with the HSCs from control mice, while SSd reversed the effect (Fig. 3C). The results from Western blot analysis further showed that CCl_4_-activated HSCs exhibited an increased LC3-II expression and decreased p62 expression compared with control, whereas SSd treatment reversed the effect (Fig. 3D–F). These data suggest that SSd inhibited CCl_4_-induced HSCs autophagy activation in vivo.

### SSd repressed HSCs autophagy activation by regulating GPER1

**Fig. 4:**
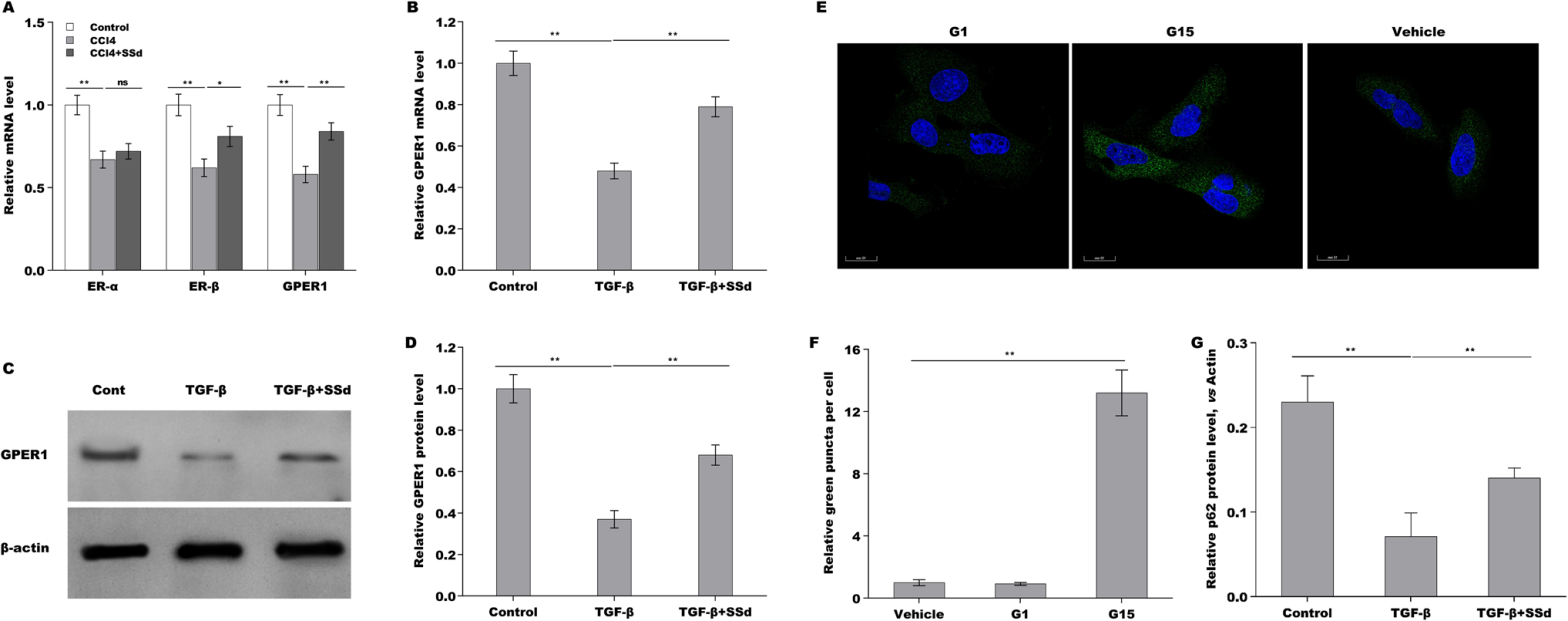
SSd repressed HSCs autophagy activation by regulating GPER1. **A** qPCR analysis of ER-α, ER-β and GPER1 mRNA expression level in primary HSCs from hepatic tissues in control mice, CCl_4_ mice and SSd-treated mice. **B** qPCR analysis of GPER1 mRNA expression level in LX-2 cells treated with TGF-β in the absence or presence of SSd. **C**, **D** Western blot analysis for GPER1 protein expression level in LX-2 cells treated with TGF-β in the absence or presence of SSd. **E**, **F** LC3 expression was detected in LX-2 cells treated with G1 (GPER1 agonist) or G15 (GPER1 antagonist) by IF assay. **G** Western blot analysis for p62 protein expression level in LX-2 cells treated with G1 or G15. **p < 0.01

**Fig. 5:**
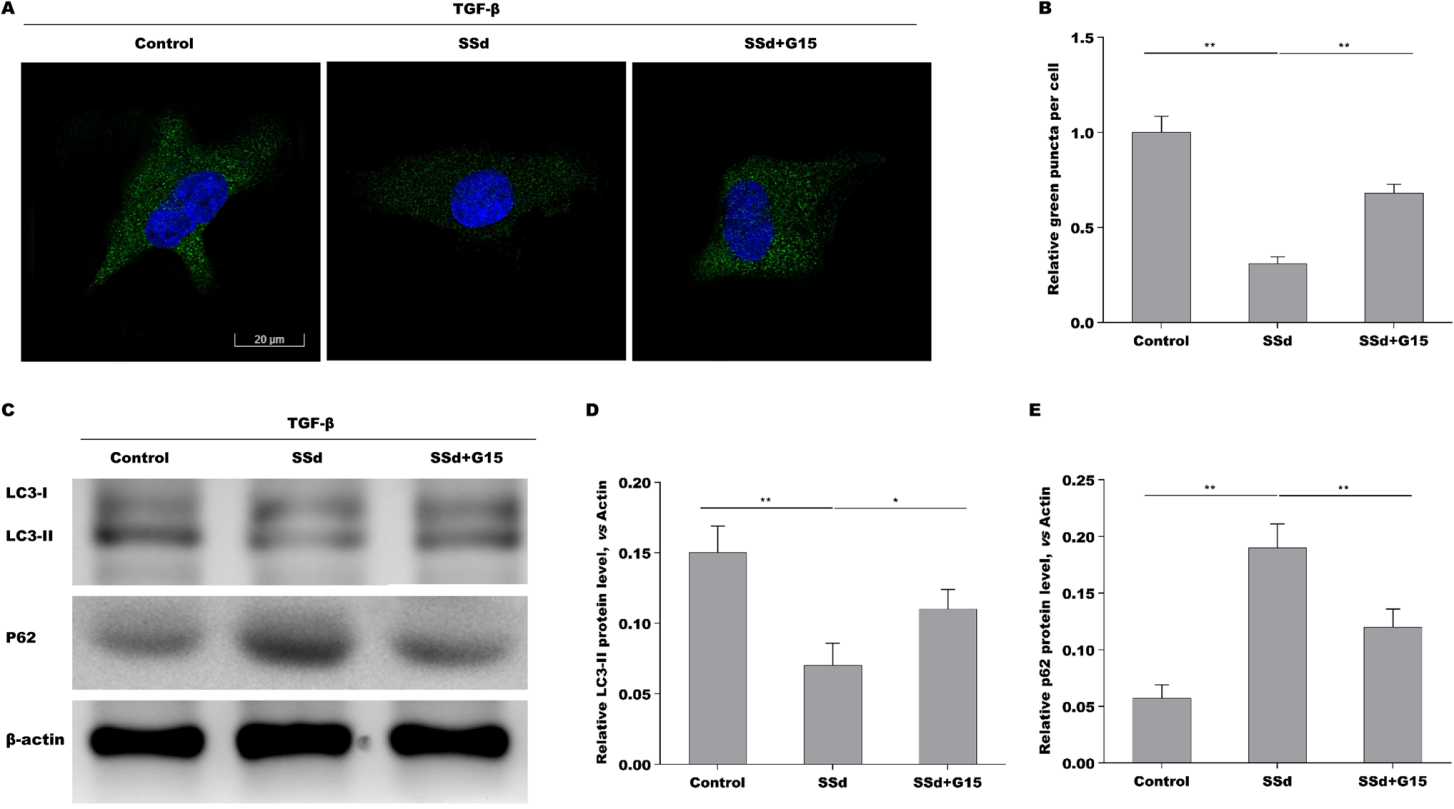
SSd repressed HSCs autophagy activation by regulating GPER1. **A**, **B** LC3 expression was detected in TGF-β-activated LX-2 cells treated with SSd in the absence or presence of G15 by IF assay. Scale bars = 20 μm for IF. **C**–**E** Western blot analysis for LC-3 (**C**, **D**) and p62 (**C**, **E**) in TGF-β-activated LX-2 cells treated with SSd in the absence or presence of G15. *p < 0.05, **p < 0.01

Our previous studies have demonstrated that SSd represses oxidative stress-induced HSCs activation by regulating estrogen receptor (ER)-β [30]. Given the important role of Estrogens in repressing HSCs activation and liver fibrosis by interacting with its nuclear receptors (ER-α, and ER-β) and membrane receptor (G protein-coupled estrogen receptor, GPER1) [31], we next investigated whether SSd exerted its anti-liver fibrotic function by regulating estrogen receptors expression. As shown in Fig. 4A, the HSCs from CCl_4_ mice exhibited a markedly decreased expression of ER-α, and ER-β, and GPER1, while SSd treatment rescued ER-β and GPER1 expression in primary HSCs. Here we focused on the correlation of SSd with GPER1 because the regulatory role of SSd in ER-β has been verified in our previous studies [30]. The results from qPCR and Western blot analysis showed that TGF-β treatment reduced the mRNA and protein expression of GPER1 in LX-2 cells, whereas SSd treatment reversed the effect (Fig. 4B–D). Then the effect of GPER1 agonist G1 and GPER1 antagonist G15 on regulating HSCs autophagy was assessed in LX-2 cells. As shown in Fig. 4E and F, G15 treatment resulted in a significant increase of LC3 puncta, though G1 did not affect LC3 puncta compared with vehicle. Western blot analysis showed that G1 upregulated p62 expression, whereas G15 repressed p62 expression (Fig. 4G). SSd decreased LC3 puncta in TGF-β-activated LX-2 cells, while G15 treatment damaged significantly the effect (Fig. 5A, B). SSd reduced LC3-II expression and enhanced p62 expression in TGF-β-activated LX-2 cells, whereas additional G15 treatment reversed the effect (Fig. 5C–E). These data demonstrate that SSd enhanced GPER1 expression in activated HSCs, and SSd repressed HSCs autophagy activation by regulating GPER1.

### SSd alleviated liver fibrosis through regulating GPER1/autophagy pathway

**Fig. 6:**
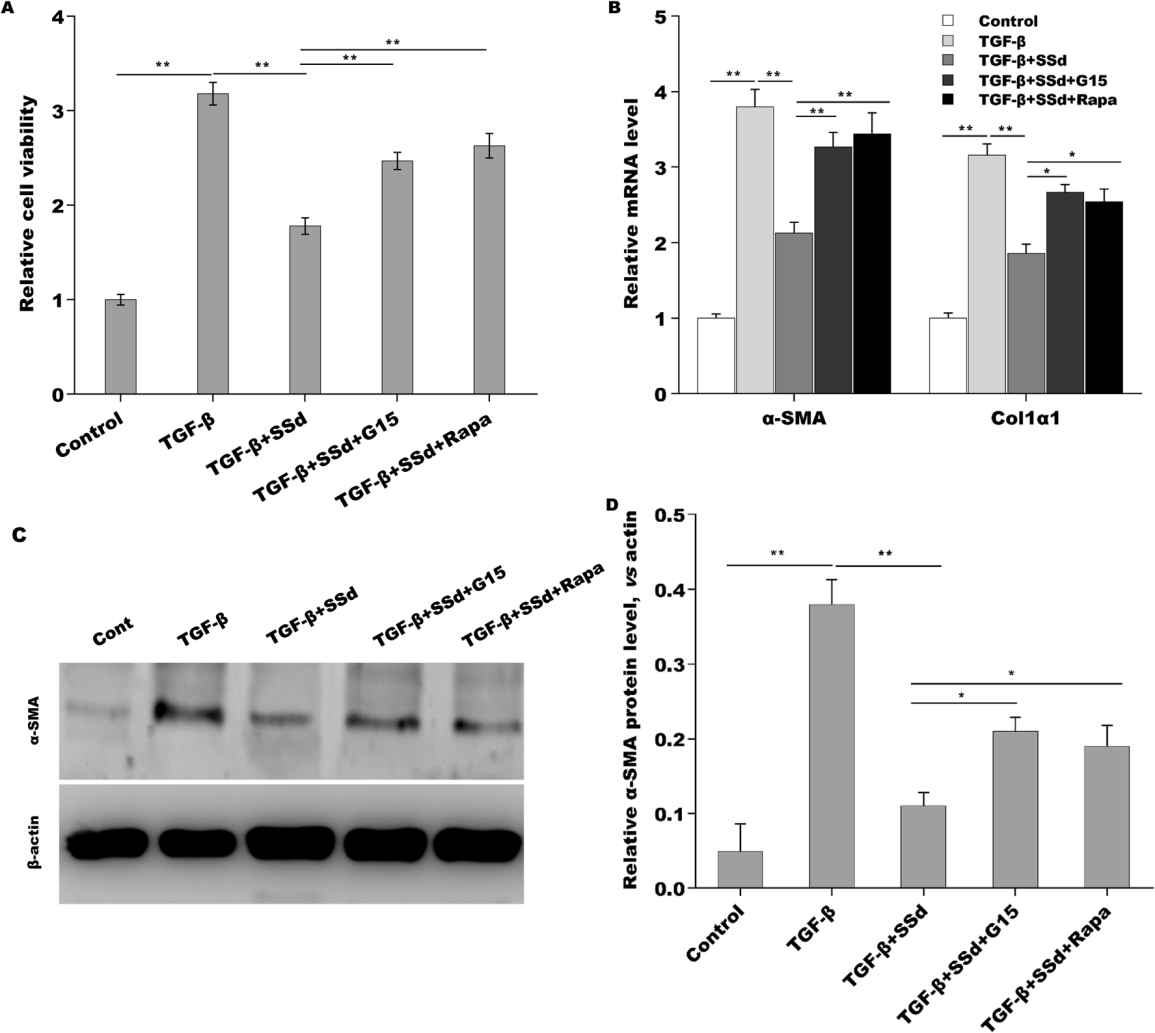
SSd alleviated liver fibrosis through regulating GPER1/autophagy pathway. **A** LX-2 cells viability was assayed using CCK-8 after TGF-β treatment in the absence or presence of SSd, G15 or Rapa. **B** qPCR analysis of α-SMA and Col1α1 mRNA expression level in TGF-β-activated LX-2 cells treated with SSd in the absence or presence of G15 or Rapa. **C**, **D** Western blot analysis for α-SMA in TGF-β-activated LX-2 cells treated with SSd in the absence or presence of G15 or Rapa. *p < 0.05, **p < 0.01

The above results showed that SSd repressed HSCs autophagy and liver fibrosis, increased GPER1 expression. Finally, we investigated whether SSd alleviated liver fibrosis through regulating GPER1/autophagy pathway. As shown in Fig. 6A, TGF-β enhanced LX-2 cells viability, whereas SSd repressed the effect. Importantly, GPER1 antagonist G15 and autophagy activator rapamycin damaged significantly the effect of SSd on suppressing LX-2 cells viability (Fig. 6A). As a result, G15 and rapamycin damaged the role of SSd in repressing Col 1 and α-SMA expression in LX-2 cells (Fig. 6B–D). Collectively, the current data demonstrate that SSd alleviated liver fibrosis through regulating GPER1/autophagy pathway.

## Discussion

Liver fibrosis is a reparative response of the liver to various chronic injuries. Inhibiting the proliferation and activation of HSCs is an effective means to prevent and treat liver fibrosis. Inactivation of HSCs autophagy contributes to repress HSCs activation and subsequent liver fibrosis [27]. Studies have shown that SSd can inhibit the activation of PSCs by activating PI3K/Akt/mTOR pathway and reduce the autophagy of PSCs, thereby improving pancreatic cancer fibrosis [32]. However, the effects of SSd on HSCs autophagy activation, liver fibrosis and the underlying mechanism are still unclear. In this study, we found that (I) SSd alleviated hepatic fibrosis and repressed autophagy activation in CCl_4_-treated mice; (II) SSd repressed CCl_4_-induced autophagy activation of HSCs in vivo; (III) SSd repressed HSCs autophagy activation by regulating GPER1; (IV) SSd alleviated liver fibrosis through regulating GPER1/autophagy pathway. The current data verify the role of SSd in repressing HSCs activation and liver fibrosis by regulating GPER1/autophagy pathway, and might be used as potential agent to treat liver fibrosis.

Autophagy is related to various organ fibroses [33]. Autophagy plays a dual role in the process of liver fibrosis. On the one hand, it activates HSCs through oxidative stress and endoplasmic reticulum stress, which leads to the occurrence and development of liver fibrosis [34]. A previous study has shown that Hepatic stellate cells acquire the energy required to activate lipid droplets by autophagic degradation, induce sustained activation of hepatic stellate cells, and promote the development of liver fibrosis [27]. On the other hand, autophagy can also play an anti-fibrotic role by regulating endothelial cells, macrophages and other roles [35]. Rapamycin, an autophagic activator, decreases the proliferation of HSCs in CCl_4_-treated mice [36]. Another research has also reported that treatment of HSCs with Phospholipase D1 (PLD1) inhibited type-I collagen accumulation through activating autophagy, which indicates PLD1 has potential anti-fibrogenic effect [37]. In this study, autophagy was increased in fibrotic liver tissues compared with control tissues, SSd treatment repressed CCl_4_-induced upregulation of BECN1 expression. In vitro cultured primary HSCs, SSd inhibited CCl_4_-induced HSCs autophagy activation. These results suggest that SSd alleviates hepatic fibrosis through suppressing the autophagy activation of HSCs.

Estrogen receptor (ER) is a glycoprotein that belongs to the nuclear receptor superfamily. ER includes two forms, ERα and ERβ, depending on the coding genes. Studies have shown that ERs also induce autophagy. For example, ERβ agonists decreased Bcl-2 level and thus activated autophagy in hormone-resistant breast cancer cells [38]. In Hodgkin lymphoma, ERβ activation decreased autophagy, suppressed proliferation, leaded to cell cycle arrest [39]. ERβ could regulate autophagy-related markers LC3 and p62 level and decreased autophagy in tumors through suppressing the PI3K/AKT/mTOR pathway and activating the AMPK pathway, as well [40]. G protein-coupled estrogen receptor 1 (GPER1) is a new ER involved in estrogen-mediated regulation of gene transcription and signaling pathways [41]. Unlike the classical ERs, which mainly act as nuclear transcription factors, GPER1 is expressed in cell membranes and cytoplasm. Recent studies reported that activation of GPER1 by G1 (a specific activator of GPER1) can inhibit autophagy via regulating the Akt-mediated pathway, thereby protecting cardiomyocytes from Ang II stimulation [42]. Studies have shown that estrogen directly interferes with the development of hepatic fibrosis through suppressing HSCs activation mediated by its receptor [43]. Our previous studies have shown that SSd, which acts as an ER regulator, can increase the expression of ERα and ERβ mRNAs in HSCs, thereby inhibiting the activation of HSCs [30]. Therefore, effective regulation of ER in HSCs is beneficial for delaying the progression of hepatic fibrosis. In this study, consistent with previous results, the HSCs from CCl_4_ mice exhibited a markedly decreased expression of ER-α, ER-β and GPER1, while SSd treatment rescued ER-β (This has been demonstrated in our previous research) and GPER1 expression in primary HSCs. To explore whether SSd represses HSCs autophagy activation by regulating GPER1. TGF-β-activated LX-2 cells was treated with SSd in the absence or presence of G15 treatment, the LC3 puncta expression was assayed by immunofluorescence and LC3 and p62 protein expressions were assayed by Western Blot analysis. Results showed that SSd decreases LC3 puncta, while G15 treatment damages significantly the effect and SSd reduces LC3-II expression and enhances p62 expression in TGF-β-activated LX-2 cells, whereas additional G15 treatment reversed the effect. These results indicate that SSd enhances GPER1 expression in activated HSCs, and SSd represses HSCs autophagy activation by regulating GPER1. Then we investigated whether SSd treatment alleviated liver fibrosis through regulating GPER1/autophagy pathway. TGF-β enhanced LX-2 cells viability, whereas SSd repressed the effect. Importantly, GPER1 antagonist G15 and autophagy activator rapamycin damaged significantly the effect of SSd on suppressing LX-2 cells viability. G15 and rapamycin damaged the role of SSd in repressing Col 1 and α-SMA expression in LX-2 cells. In summary, the current data demonstrate that SSd alleviated liver fibrosis through regulating GPER1/autophagy pathway.

## Data Availability

The datasets used and/or analyzed during the current study are available from the corresponding author on reasonable request.
